# Geographic Characteristics and Mortality Profiles in the JPHC Study

**DOI:** 10.2188/jea.11.6sup_8

**Published:** 2007-11-30

**Authors:** Seiichiro Yamamoto, Shaw Watanabe

**Affiliations:** 1 Cancer Information and Epidemiology Division, National Cancer Center Research Institute.; 2 Department of Applied Bioscience, Tokyo University of Agriculture.

**Keywords:** mortality, standardized mortality ratio, demography, prospective cohort study

## Abstract

The study areas of the Japan Public Health Center-based Prospective Study on Cancer and Cardiovascular Diseases (JPHC Study) are distributed throughout Japan and represent both rural and urban communities. These geographical differences yield considerable difference in population, culture, and lifestyle. The mortality rates in the study areas were apparently influenced by these factors. The pattern of standardized mortality ratios (SMRs) for all causes of death (cancer, heart diseases, ischemic heart disease, and cerebrovascular diseases) in each area was different. Age-standardized site-specific cancer mortality rates showed large variation even when compared with corresponding figures of prefectures in Japan. The areas of the JPHC study showed different patterns of SMR for major causes of death. The differences in site-specific cancer mortality rates for most of the sites are sufficient for epidemiological analyses.

## INTRODUCTION

The Japan Public Health Center-based Prospective Study on Cancer and Cardiovascular Diseases (JPHC) Study encompasses the prefectures with the highest and lowest mortality rates. Nagano and Okinawa showed the longest life expectancy in Japan, whereas Osaka had the shortest. The districts in cohort I (Ninohe, Yokote, Saku, Tokyo, and Ishikawa) are mostly in central and northeastern Japan. The addition of cohort II (Kashiwazaki, Kasama, Suita, Tosayamada, Arikawa, and Miyako), which are mostly located in western Japan, made the study area representative of Japan, covering both the northern and southern regions. Variation in mortality and baseline characteristics between the different study areas were large enough to investigate relationships.

## MATERIALS AND METHODS

Information on population, size of districts, and composition of industries in each study area was obtained from the 1990 Population Census of Japan^1^^)^. Using 1988-1992 vital statistics^2^^)^, standardized mortality ratios (SMRs) of the study areas were calculated for all causes of deaths, deaths due to cerebrovascular diseases (430-438), malignant neoplasm (140-208), cardiovascular diseases (393-398, 410-414, 415-429), and ischemic heart diseases (410-414). The values in parenthesis are corresponding codes in the International Classification of Diseases (ICD9)^3^^)^. The formula are as follows:

$$SMR=\frac{\text{Number of observed deaths due to causes of interest in the area}}{\text{Number of expected deaths due to causes of interest in the area}}\times 100$$

where number of observed deaths include those from 1 January 1988 to 31 December 1992. Number of expected deaths are calculated as:

$$\begin{array}{rl} & \text{Number of expected deaths}=\\ & \;5\times \sum_{\text{age}}\text{(age-specific national mortality rate in 1992)}\times \text{(age-specific population in the area)} \end{array}$$

Observed numbers of deaths and age-specific mortality rates for the given areas are from vital statistics, and age-specific population figures are from the 1990 population census of Japan.

For site-specific cancer mortality rates, age-standardized mortality rates (SRs) and SMRs were calculated. The formula of SR for each site is as follows:

$$SR=\sum_{\text{1980-1990}}\sum_{\text{age}}\text{age-specific mortality rate in the area}\times \frac{\text{age-specific number of standard population}}{\text{total number of standard population}}$$

where age-specific mortality rates for given areas are calculated from vital statistics, and the 1985 model population of Japan is used as the age-specific standard population. SRs were also calculated using world population as the age-specific standard population.

SMRs for each site are calculated as follows:

$$SMR=\frac{\text{Number of site-specific observed deaths in the area}}{\text{Number of site-specific expected deaths in the area}}\times 100$$

where the number of site-specific observed deaths includes deaths from 1 January 1980 to 31 December 1990. Number of site-specific expected deaths was calculated as follows:

$$\begin{array}{rl} & \text{Number of expected deaths}=\\ & \;\sum_{\text{1980-1990}}\sum_{\text{age}}\text{(age-specific national mortality rate}\\ & \;\;\;\text{ from 1 January to 31 December each year)}\\ & \;\times \text{(area-age specific population estimated from population census)} \end{array}$$

The observed number of site-specific deaths and age-specific mortality rates for each area were obtained from the vital statistics. Age-specific population figures came from the Population Censuses of 1980, 1985, and 1990 or were estimated by extrapolation using pre- and postnational census figures for the year when a census was not conducted.

Area-specific cumulative risk and cumulative rate from age 0-74 for site-specific cancer mortality were also calculated, using the following formulas:

$$\text{cumulative rate}=5\sum_{\text{age}}\text{age-specific mortality rate in the area}$$

cumulative risk=1−cumulative rate

Classification of cancer followed the ICD9, where 140-208 represent all cancers: esophagus, 150; stomach, 151; colon, 153; rectal, 154; liver, 155, 199.1c; biliary tract, 156; pancreas, 157; lung, 162; skin 172, 173; breast, 174, 175; uterine cancer, 179-182; leukemia, 204-208; and malignant lymphoma, 200-203. In this paper, SMR > 110 are considered high scores and SMR < 90 are considered low.

## Results

Population numbers; size of areas; and proportion of primary, secondary, and tertiary industries in each area are shown in Table 1. Tertiary industry accounted for more than 60% in Katsushika and Suita, but even in some rural areas it represented more than 50%. Primary industry exceeded 50% in only Gusukube in the Miyako public health center area and a few villages in the Saku public health center area.

**Table 1.** Backgroud profile of study area.

					Industrial classification*		

Health Center	Prefecture	Local municipality	Population	Area (Km^2^)	Primary	Secondary	Tertiary

Ninohe	Iwate	Ninohe city	28,501	240.6	23.5	24.6	44.9
		Karumai town	13,036	245.7	42.2	27.6	30.2
		total	41,537	486.4	-	-	-
Yokote	Akita	Yokote city	41,623	110.6	14.1	25.8	60.1
		Omonogawa town	12,439	73.6	33.9	34.3	31.8
		total	54,062	184.2	-	-	-
Saku	Nagano	Usuda town	15,982	83.2	14.0	41.7	44.3
		Saku town	9,104	122.1	21.3	41.9	36.8
		Yachiho village	4,940	66.0	26.2	39.1	34.7
		Koumi town	6,583	114.2	25.9	29.5	44.6
		Minamiaiki village	1,373	66.0	45.0	25.7	29.3
		Kitaaiki village	1,081	56.3	34.9	39.0	26.1
		Minamimaki village	3,631	133.1	53.8	9.0	37.2
		Kawakami town	4,839	209.6	65.0	9.1	25.9
		total	47,533	850.5	-	-	-
Ishikawa	Okinawa	Gushikawa city	57,932	31.0	8.2	22.9	68.9
		Onna town	9,052	50.7	20.3	16.4	63.3
		total	66,984	81.7	-	-	-
Katsushika	Tokyo	Katsushika ward	422,796	34.8	0.3	36.9	62.8

Kasama	Ibaraki	Tomobe town	33,241	58.7	10.7	31.8	57.5
		Iwase town	23,637	87.2	13.1	44.0	42.9
		total	56,878	145.9	-	-	-
Kashiwazaki	Niigata	Oguni town	8,106	86.2	24.6	45.7	29.7
Tosayamada	Kochi	Kagami town	6,297	58.9	42.7	19.6	37.7
		Noichi town	15,298	22.9	23.3	19.5	57.2
		total	21,595	81.8	-	-	-
Arikawa	Nagasaki	Uku town	4,468	26.4	37.6	22.1	40.3
		Ojika town	4,310	25.4	48.3	10.7	41.0
		Shinuonome town	5,353	25.2	27.4	20.8	51.8
		Arikawa town	8,598	57.0	25.2	18.6	56.2
		Kamigoto town	8,100	55.7	25.8	18.4	55.8
		Narao town	4,013	15.4	31.6	18.5	49.9
		total	34,842	205.0	-	-	-
Miyako	Okinawa	Hirara city	34,314	64.6	17.8	18.9	63.3
		Gusukube town	8,270	57.6	61.8	12.2	26.0
		total	42,584	122.2	-	-	-
Suita	Osaka	Suita city	330,391	36.1	0.2	27.5	72.3


*Industrial classification ^1)^

primary (agriculture, forestry, fisheries)

secondary (mining, construction, manufacturing),

tertiary [electricity, gas, heat supply and water, transport and communication, wholesale and retail trade and eating and drinking places, financing and insurance, real estate, services, government (not elsewhere clasified)]

**Figure 1.**
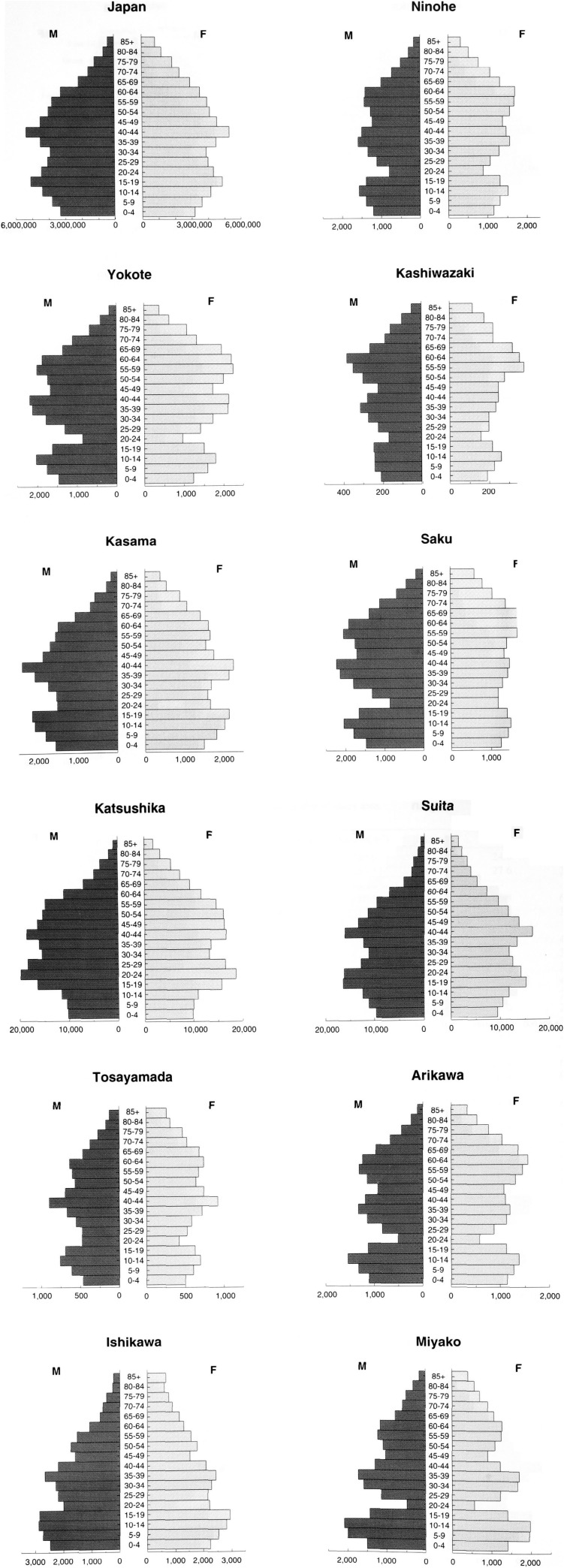
Population pyramid

Population pyramids in each area are shown in Fig. 1. The proportion of younger people (20-29 age category) was large in Katsushika (Tokyo) and Suita (Osaka). In contrast, the aged category (more than 65) was prominent in Kashiwazaki (Niigata), Saku (Nagano), and Tosayamada (Kochi). A deficit in the age 20-24 category, especially males, suggested their moving toward large metropolitan cities.

**Figure 2.**
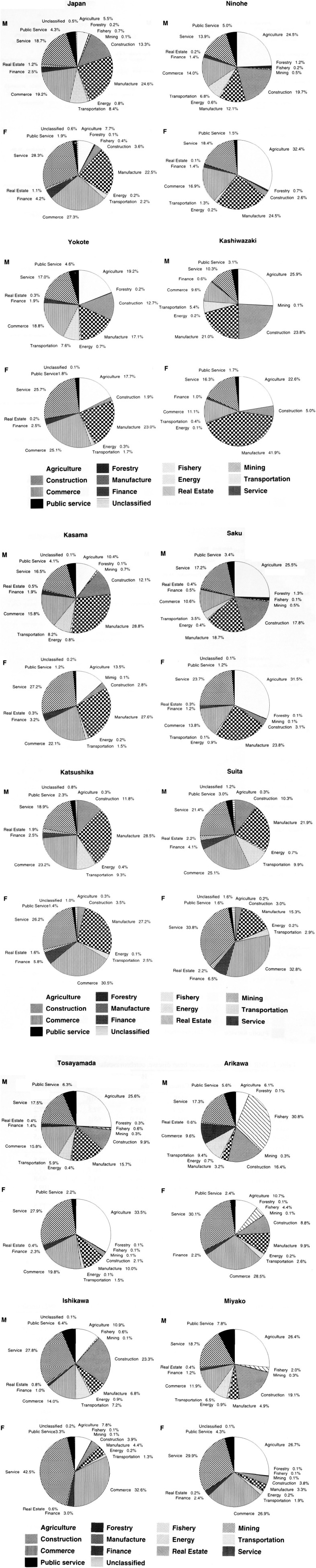
Occupation

The number of workers according to industry is shown in Fig. 2. The urban areas of Katsushika and Suita showed a very low proportion of farmers. Instead, manufacturing, trade, and the service industries were dominant. Agriculture accounted for nearly one-fourth of the jobs in Ninohe, Yokote, Kashiwazaki, Saku, Tosayamada, and Miyako, and the fishing industry is prominent in Arikawa. Thus, each public health center district has rather characteristic industrial features.

As for the mortality in cohort I areas, in Ninohe, SMRs for all causes (male), heart disease (male), and cerebrovascular disease (both sexes) were high (Tables 2 and 3, Fig. 3), whereas SMRs for ischemic heart disease (male) and cancer (female) were low. In Yokote, the SMR for cerebrovascular disease (female) was high, whereas SMRs for heart disease (female) and ischemic heart disease (both sexes) were low. In Saku, SMRs for all causes (cancer, heart disease, ischemic heart disease, and cerebrovascular diseases) were all low in both sexes. In Katsushika, SMRs for all causes (female), cancer (female), ischemic heart disease (both sexes), and cerebrovascular disease (both sexes) were high. In Ishikawa, SMRs for all causes were low in both sexes.

**Table 2.** SMR for all causes, cancer heart disease, cerebrovascular disease, 1988-1992 (male).

	All causes	Cancer	Heart disease	Ischaemic heart disease	Cerebro- vascular disease

	SMR	SMR	SMR	SMR	SMR
Ninohe	119.0	92.7	129.2	77.5	127.2
Yokote	105.8	104.7	93.0	68.1	125.7
Saku	89.0	89.4	66.7	44.8	84.2
Katsushika	109.1	108.2	102.6	170.8	121.5
Ishikawa	86.1	84.6	72.5	69.7	63.6

Kasama	101.4	89.2	101.9	89.4	108.5
Kashiwazaki	81.6	69.2	74.2	66.7	73.7
Tosayamada	95.6	91.9	99.6	74.5	92.8
Arikawa	116.9	136.0	105.2	101.2	109.8
Miyako	95.5	92.9	68.5	59.0	68.1
Suita	93.2	105.0	94.7	98.4	72.5

**Table 3.** SMR for all causes, cancer heart disease, cerebrovascular disease, 1988-1992 (female).

	All causes	Cancer	Heart disease	Ischaemic heart disease	Cerebro- vascular disease

	SMR	SMR	SMR	SMR	SMR
Ninohe	102.3	87.0	104.1	91.6	118.1
Yokote	101.3	107.0	87.4	65.0	141.4
Saku	87.5	88.0	68.9	52.6	88.5
Katsushika	111.4	113.0	107.8	178.3	117.2
Ishikawa	77.1	82.1	70.7	61.6	42.7

Kasama	98.9	104.0	77.8	78.4	137.6
Kashiwazaki	89.4	94.4	49.5	57.8	100.7
Tosayamada	102.9	92.0	104.2	80.3	94.1
Arikawa	106.9	124.6	86.5	75.8	97.1
Miyako	85.9	87.4	51.8	41.0	57.6
Suita	102.6	117.4	105.7	99.9	74.6

**Figure 3-1.**
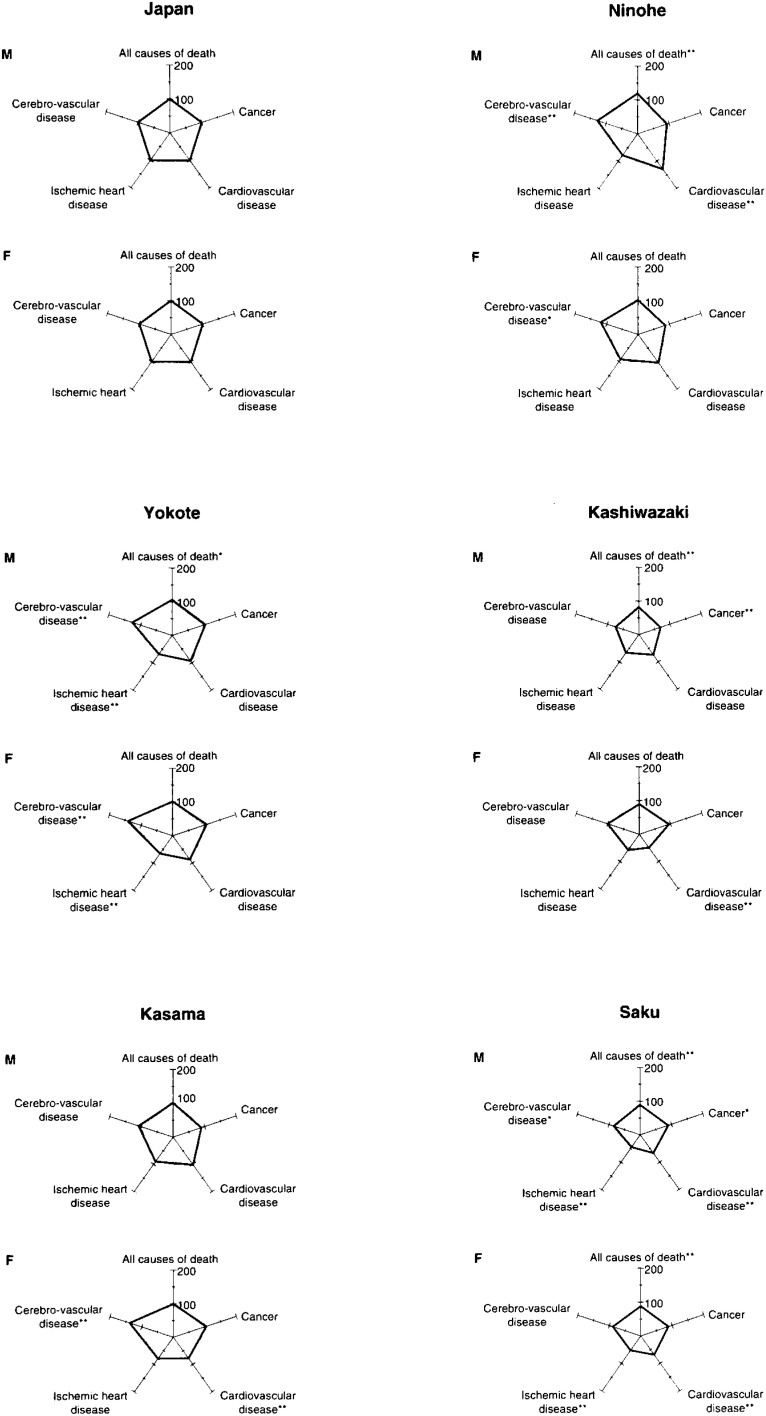
SMR by major causes of deaths in the JPHC Study Cohort I Study Area (1988-1992)

**Figure 3-2.**
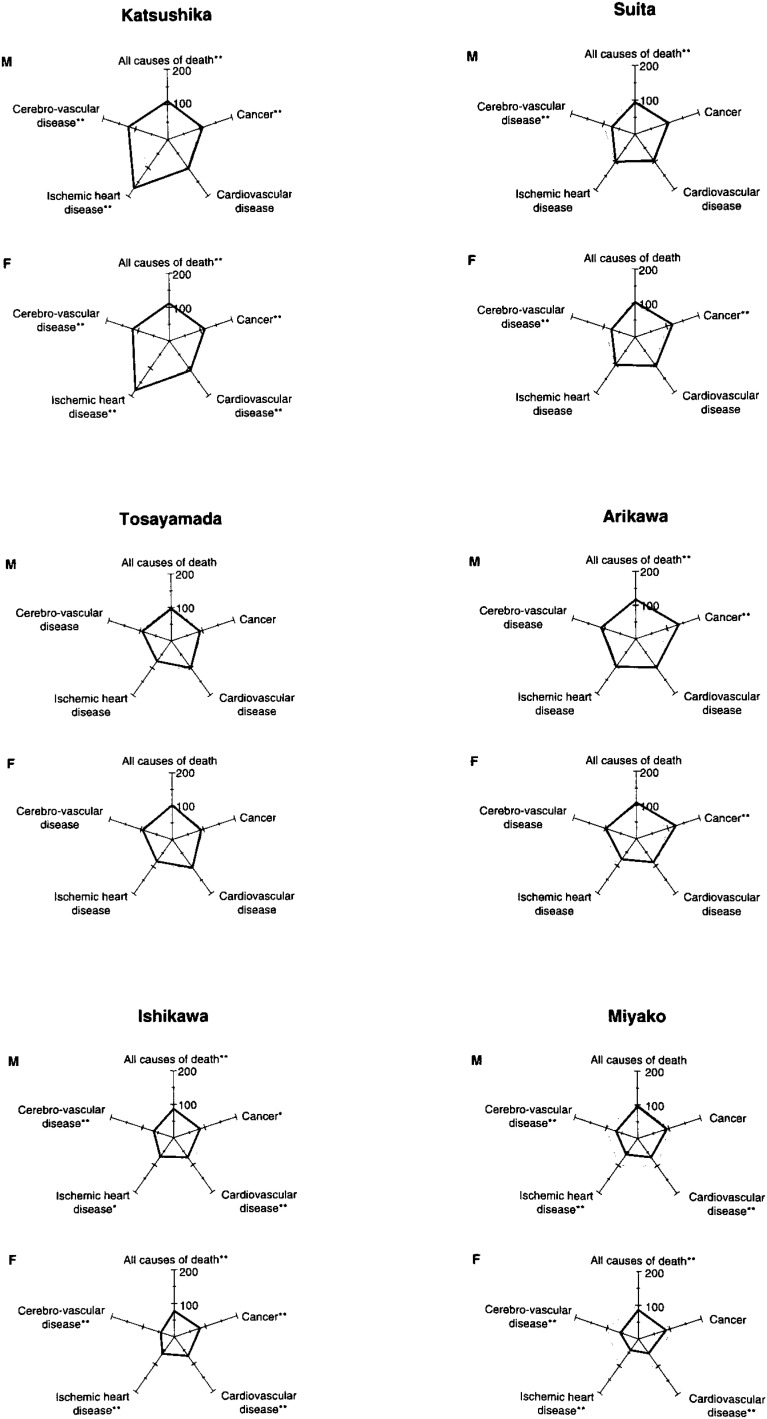
SMR by major causes of deaths in the JPHC Sutudy Cohort II Study Area (1988-1992)

As for the areas in cohort II, in Kasama, SMR for cerebrovascular disease (female) was low, as well as SMRs for cancer (male), heart disease (female), and ischemic heart disease (both sexes). In Kashiwazaki, SMRs for all causes (both sexes), cancer (male), heart disease (both sexes), ischemic heart disease (both sexes), and cerebrovascular disease (male) were low. In Tosayamada, SMRs for ischemic heart disease (both sexes) were low.

In Arikawa, SMRs for all causes (male) and cancer (both sexes) were high. SMRs for heart disease (female) and ischemic heart disease (female) were low. In Miyako, SMRs for all causes (female), cancer (female), heart disease (both sexes), ischemic heart disease (both sexes), and cerebrovascular disease (both sexes) were low. In Suita, SMR for cancer (female) was high, although SMR for cerebrovascular disease (female) was low.

Age-standardized site-specific cancer mortality rates are shown in Tables 4 and 5, using the 1985 model population of Japan as the standard, and in Tables 6 and 7, using the world population as the standard. Cancer mortality rates of males were more than twice those of females at most of the sites. Highest and lowest mortality rates for each site were as follows: for stomach, Yokote (male) and Kasama (female) were highest and Ishikawa (both sexes) was lowest; for lung, Arikawa (male) and Ishikawa (female) were highest and Kashiwazaki (male) and Ninohe (female) were lowest; for liver, Arikawa (both sexes) was highest and Ishikawa (both sexes) was lowest; for pancreas, Ninohe (male) and Tosayamada (female) were highest and Tosayamada (male) and Ishikawa (female) were lowest; for colon, Suita (male) and Kashiwazaki (female) were highest and Kashiwazaki (male) and Ishikawa (female) were lowest; for esophagus, Miyako (male) and Arikawa (female) were highest and Ninohe (male) and Miyako (female) were lowest;for rectum, Kasama (male) and Kashiwazaki (female) were highest and Ishikawa (male) and Tosayamada (female) were lowest; for biliary tract, Arikawa (male) and Kasama (female) were highest and Ishikawa (both sexes) was lowest; for leukemia, Ishikawa (male) and Kashiwazaki (female) were highest and Ninohe (both sexes) was lowest; for malignant lymphoma, Arikawa (both sexes) was highest and Kashiwazaki (both sexes) was lowest;for skin, Kashiwazaki (male) and Tosayamada (female) were highest and Saku (male) and Kashiwazaki (female) were lowest;for prostate, Ninohe was highest and Kashiwazaki was lowest;for breast, Katsushika was highest and Miyako was lowest;for uterus, Kasama was highest and Kashiwazaki was lowest.

**Table 4.** Area-specific age-standardized mortality rate for male (/100,000) (Japan 1985 model population as standard).

HC	All cancer	Stomach	Lung	Liver	Pancreas	Colon	Esophagus	Rectum	Biliary tract	Leukemia	Lymphoma	Prostate	Skin
Ninohe	197.6	46.8	44.5	13.4	14.7	10.8	4.0	12.2	11.1	3.8	3.5	9.2	0.9
Yokote	245.7	85.2	45.2	14.0	12.3	12.4	20.8	6.5	11.8	6.4	2.0	5.2	0.7
Saku	211.6	62.5	38.8	10.8	13.4	12.7	13.6	10.7	8.3	7.4	7.4	7.2	0.3
Katsushika	255.4	75.2	47.1	28.4	12.9	15.4	14.6	10.2	7.1	7.0	4.9	7.0	1.2
Ishikawa	185.5	27.6	46.4	10.4	6.9	13.7	17.9	5.0	3.3	15.9	7.9	5.0	0.9
Kasama	233.1	78.7	37.5	18.6	10.8	10.7	17.1	12.2	7.0	5.3	3.6	6.3	0.9
Kashiwazaki	179.9	64.5	30.2	13.4	6.5	4.6	12.5	7.5	5.8	7.4	1.5	2.8	4.9
Tosayamada	202.3	57.6	41.6	22.6	5.1	7.3	4.2	7.9	6.8	11.8	5.2	7.7	1.1
Arikawa	318.1	65.2	62.2	54.8	14.3	11.5	13.6	9.5	20.4	12.2	14.2	5.0	0.5
Miyako	224.5	45.3	45.5	20.2	11.8	13.7	21.6	9.4	7.6	11.0	5.4	3.7	1.9
Suita	247.9	60.8	49.3	40.8	12.4	15.4	8.4	9.5	8.7	7.8	5.5	5.8	0.3

Japan	214.5	58.3	41.4	26.7	11.5	11.0	9.9	9.0	7.8	6.7	5.4	5.4	0.7
maximum prefecture	259.5	77.8	53.9	47.4	16.7	13.6	17.5	11.1	-	10.5	-	-	-
minimum prefecture	183.6	28.8	31.5	14.5	8.3	6.9	3.5	6.4	-	3.5	-	-	-
maximum*	318.1	85.2	62.2	54.8	14.7	15.4	21.6	12.2	20.4	15.9	14.2	9.2	4.9
Minimum**	179.9	27.6	30.2	10.4	5.1	4.6	4.0	5.0	3.3	3.8	1.5	2.8	0.3
difference***	138.2	57.7	32.0	44.4	9.6	10.8	17.6	7.2	17.0	12.1	12.7	6.4	4.7
ratio****	1.8	3.1	2.1	5.3	2.9	3.3	5.4	2.4	6.1	4.2	9.5	3.3	18.3

*maximum of age-adjusted rate between areas. **minimum of age-adjusted rate between areas.

***difference between maximum and minimum rates. ****ratio of maximum to minimum rates.

**Table 5.** Area-specific age-standardized mortality rate for female (/100,000) (Japan 1985 model population as standard).

HC	All cancer	Stomach	Lung	Colon	Liver	Breast	Biliary tract	Uterine	Pancreas	Rectum	Leukemia	Lymphoma	Esophagus	Skin
Ninohe	94.4	15.2	10.2	6.7	5.4	7.4	7.2	4.9	7.8	4.7	1.6	4.3	0.9	0.6
Yokote	128.7	35.2	11.7	11.9	4.3	5.0	10.6	4.9	8.4	6.1	4.0	3.5	1.3	0.2
Saku	112.0	27.3	12.4	10.4	7.8	6.9	8.2	6.6	5.4	5.5	3.8	3.3	1.1	0.3
Katsushika	133.0	32.0	14.6	10.3	8.5	11.1	8.4	8.5	8.0	5.4	3.9	3.2	2.4	0.4
Ishikawa	97.6	12.3	17.1	4.7	4.3	4.6	6.4	12.1	3.5	4.5	9.0	7.1	2.3	0.2
Kasama	128.7	39.4	10.6	8.4	6.9	5.1	11.6	13.2	4.9	5.1	3.3	3.0	1.1	0.3
Kashiwazaki	138.0	36.9	12.5	13.1	5.0	10.1	8.5	1.4	10.7	12.7	9.1	2.5	0.9	0.0
Tosayamada	127.4	29.9	11.7	9.2	6.0	7.4	6.4	9.8	11.6	3.3	6.1	5.7	0.9	1.0
Arikawa	138.5	25.3	16.4	9.6	14.7	7.4	9.5	7.4	8.1	5.4	6.3	7.4	3.0	0.7
Miyako	101.2	13.3	16.5	6.9	7.8	3.7	11.3	6.2	4.9	4.3	4.7	6.8	0.8	0.6
Suita	130.6	27.7	15.4	9.0	10.7	10.4	7.3	9.1	7.5	4.9	4.1	4.3	1.7	0.4

Japan	112.4	26.8	11.3	8.4	8.0	7.7	7.7	7.2	6.6	4.9	3.7	3.4	1.7	0.4
maximum*	138.5	39.4	17.1	13.1	14.7	11.1	11.6	13.2	11.6	12.7	9.1	7.4	3.0	1.0
minimum**	94.4	12.3	10.2	4.7	4.3	3.7	6.4	1.4	3.5	3.3	1.6	2.5	0.8	0.0
difference***	44.2	27.1	6.9	8.5	10.5	7.4	5.3	11.8	8.1	9.3	7.5	4.9	2.2	1.0
ratio****	1.5	3.2	1.7	2.8	3.5	3.0	1.8	9.6	3.3	3.8	5.8	3.0	3.8	-

*maximum of age-adjusted rate between areas. **minimum of age-adjusted rate between areas.

***difference between maximum and minimum rates. ****ratio of maximum to minimum rates.

**Table 6.** Area-specific age-standardized mortality rate for male (/100,000) (world population as standard).

HC	All cancer	Stomach	Lung	Liver	Pancreas	Colon	Esophagus	Rectum	Biliary tract	Leukemia	Lymphoma	Prostate	Skin
Ninohe	138.3	33.2	30.6	9.4	10.8	7.8	2.9	8.3	8.1	2.5	2.5	5.5	0.7
Yokote	171.5	59.6	31.4	10.2	8.5	8.5	14.8	4.5	8.3	4.6	1.2	2.8	0.6
Saku	145.5	42.8	25.9	7.3	8.9	8.4	9.2	7.0	5.5	5.7	6.9	4.7	0.2
Katsushika	177.6	51.7	31.8	20.7	9.0	10.3	10.4	7.3	4.6	5.1	4.3	4.3	0.8
Ishikawa	129.7	19.5	30.7	7.8	4.6	10.0	11.9	3.5	2.3	11.6	5.6	3.4	0.7
Kasama	162.7	54.7	25.6	13.2	7.9	7.5	12.1	8.2	5.0	3.4	3.5	4.0	0.5
Kashiwazaki	125.7	44.0	19.9	11.5	4.7	3.6	9.2	5.0	3.4	4.9	1.3	1.3	4.1
Tosayamada	143.2	41.1	28.1	15.9	3.5	6.0	2.6	5.4	4.5	9.8	4.4	4.6	0.9
Arikawa	222.4	45.1	41.7	40.6	9.9	7.9	9.6	6.9	13.0	8.7	11.3	3.1	0.2
Miyako	161.7	31.8	31.5	15.1	8.5	10.0	15.6	7.0	5.4	8.4	5.0	2.6	0.9
Suita	172.7	41.9	33.1	29.4	8.4	10.6	6.0	6.8	5.7	5.6	4.9	3.7	0.2

Japan	149.5	40.2	27.9	19.4	8.0	7.6	6.9	6.2	5.3	4.9	4.5	3.4	0.5

**Table 7.** Area-specific age-standardized mortality rate for female (/100,000) (world population as standard).

HC	All cancer	Stomach	Lung	Colon	Liver	Breast	Biliary tract	Uterine	Pancreas	Rectum	Leukemia	Lymphoma	Esophagus	Skin
Ninohe	66.3	10.6	7.0	4.2	3.5	5.6	4.7	3.7	5.5	3.0	1.2	3.4	0.5	0.4
Yokote	90.5	23.8	8.1	8.5	2.9	3.7	7.2	3.4	5.5	4.4	2.8	3.1	0.8	0.2
Saku	78.3	17.9	8.3	7.1	5.7	5.1	5.4	4.8	3.6	3.9	3.1	2.8	0.8	0.2
Katsushika	93.7	22.3	9.7	6.9	5.8	8.3	5.6	6.4	5.4	3.7	3.0	2.6	1.7	0.2
Ishikawa	69.1	8.7	11.5	3.1	2.9	3.6	3.8	9.0	2.4	3.2	6.6	5.7	1.7	0.1
Kasama	91.7	28.9	7.3	6.1	4.6	3.9	7.8	9.5	3.2	3.9	2.2	2.4	0.8	0.2
Kashiwazaki	98.3	25.6	8.7	8.9	3.0	8.1	5.5	1.2	8.0	9.8	6.2	3.2	0.5	0.0
Tosayamada	92.3	20.4	8.7	5.8	3.8	6.0	4.1	7.2	7.9	2.2	3.6	5.6	0.6	0.4
Arikawa	99.6	18.5	10.8	6.3	10.8	5.7	6.3	5.2	5.9	3.9	4.3	5.8	2.2	0.5
Miyako	70.2	9.2	10.9	4.6	5.1	2.9	7.6	4.3	3.1	3.0	3.4	5.2	0.6	0.5
Suita	92.0	19.2	10.4	6.1	7.5	8.0	5.0	6.5	4.8	3.3	3.0	3.7	1.1	0.3

Japan	79.7	18.68	7.71	5.8	5.59	5.9	5.22	5.17	4.52	3.43	2.7	2.89	1.12	0.29

Area-specific cumulative risks and rates for site-specific cancer mortality are shown in Tables 8 and 9 (males) and Tables 10 and 11 (females). Patterns similar to age-adjusted mortality rates were observed..

**Table 8.** Area-specific cumulative risk (0-74) for male.

HC	All cancer	Stomach	Lung	Liver	Pancreas	Colon	Esophagus	Rectum	Biliary tract	Leukemia	Lymphoma	Prostate	Skin
Ninohe	0.149	0.041	0.036	0.010	0.014	0.009	0.003	0.008	0.013	0.002	0.003	0.004	0.001
Yokote	0.180	0.064	0.041	0.013	0.010	0.008	0.019	0.006	0.010	0.004	0.001	0.001	0.001
Saku	0.140	0.047	0.026	0.007	0.009	0.008	0.010	0.007	0.006	0.006	0.005	0.005	0.000
Katsushika	0.181	0.056	0.037	0.024	0.011	0.011	0.012	0.008	0.005	0.005	0.004	0.003	0.001
Ishikawa	0.139	0.023	0.036	0.011	0.003	0.014	0.013	0.005	0.002	0.013	0.004	0.002	0.001
Kasama	0.172	0.064	0.031	0.015	0.010	0.008	0.015	0.008	0.005	0.003	0.003	0.005	0.001
Kashiwazaki	0.122	0.038	0.020	0.016	0.004	0.006	0.010	0.004	0.005	0.006	0.002	0.000	0.003
Tosayamada	0.156	0.052	0.032	0.018	0.007	0.007	0.004	0.008	0.006	0.011	0.003	0.001	0.001
Arikawa	0.221	0.046	0.048	0.048	0.013	0.009	0.012	0.007	0.014	0.011	0.013	0.001	0.000
Miyako	0.173	0.041	0.038	0.018	0.011	0.010	0.019	0.008	0.007	0.010	0.004	0.002	0.000
Suita	0.182	0.046	0.040	0.036	0.009	0.012	0.007	0.008	0.006	0.007	0.004	0.004	0.000

**Table 9.** Area-specific cumulative rate (0-74) for male.

HC	All cancer	Stomach	Lung	Liver	Pancreas	Colon	Esophagus	Rectum	Biliary tract	Leukemia	Lymphoma	Prostate	Skin
Ninohe	0.161	0.042	0.037	0.010	0.014	0.009	0.003	0.008	0.013	0.002	0.003	0.004	0.001
Yokote	0.199	0.066	0.041	0.013	0.010	0.008	0.019	0.006	0.010	0.004	0.001	0.001	0.001
Saku	0.151	0.048	0.026	0.007	0.009	0.008	0.010	0.007	0.006	0.006	0.005	0.005	0.000
Katsushika	0.199	0.057	0.038	0.025	0.011	0.011	0.012	0.009	0.005	0.005	0.004	0.003	0.001
Ishikawa	0.149	0.023	0.037	0.011	0.003	0.014	0.013	0.005	0.002	0.013	0.004	0.002	0.001
Kasama	0.188	0.066	0.031	0.015	0.010	0.008	0.015	0.008	0.005	0.003	0.003	0.005	0.001
Kashiwazaki	0.130	0.039	0.020	0.016	0.004	0.006	0.010	0.004	0.005	0.006	0.002	0.000	0.003
Tosayamada	0.170	0.054	0.033	0.018	0.007	0.007	0.004	0.008	0.006	0.011	0.003	0.001	0.001
Arikawa	0.250	0.047	0.049	0.049	0.013	0.009	0.012	0.007	0.014	0.011	0.013	0.001	0.000
Miyako	0.190	0.042	0.038	0.019	0.011	0.010	0.020	0.008	0.007	0.010	0.004	0.002	0.000
Suita	0.201	0.047	0.040	0.036	0.009	0.012	0.007	0.008	0.006	0.007	0.004	0.004	0.000

**Table 10.** Area-specific cumulative risk (0-74) for female.

HC	All cancer	Stomach	Lung	Colon	Liver	Breast	Biliary tract	Uterrine	Pancreas	Rectum	Leukemia	Lymphoma	Esophagus	Skin
Ninohe	0.072	0.012	0.009	0.004	0.004	0.006	0.006	0.005	0.007	0.003	0.001	0.003	0.000	0.001
Yokote	0.094	0.022	0.009	0.010	0.003	0.004	0.008	0.004	0.006	0.005	0.004	0.003	0.001	0.000
Saku	0.078	0.014	0.009	0.007	0.007	0.005	0.006	0.005	0.006	0.004	0.003	0.003	0.001	0.000
Katsushika	0.097	0.023	0.010	0.007	0.006	0.009	0.006	0.008	0.007	0.004	0.003	0.002	0.002	0.000
Ishikawa	0.072	0.009	0.011	0.003	0.004	0.004	0.004	0.011	0.003	0.003	0.008	0.006	0.002	0.000
Kasama	0.099	0.031	0.008	0.008	0.006	0.004	0.010	0.010	0.004	0.003	0.003	0.002	0.001	0.001
Kashiwazaki	0.100	0.027	0.008	0.010	0.004	0.008	0.007	0.001	0.010	0.010	0.006	0.002	0.000	0.000
Tosayamada	0.090	0.023	0.010	0.005	0.004	0.006	0.003	0.009	0.008	0.003	0.003	0.004	0.001	0.000
Arikawa	0.100	0.018	0.011	0.006	0.014	0.006	0.007	0.005	0.005	0.004	0.005	0.006	0.003	0.001
Miyako	0.076	0.011	0.012	0.005	0.006	0.004	0.009	0.004	0.003	0.003	0.004	0.006	0.001	0.000
Suita	0.097	0.021	0.011	0.007	0.009	0.009	0.006	0.008	0.004	0.003	0.003	0.004	0.001	0.000

**Table 11.** Area-specific cumulative rate (0-74) for female.

HC	All cancer	Stomach	Lung	Colon	Liver	Breast	Biliary tract	Uterine	Pancreas	Rectum	Leukemia	Lymphoma	Esophagus	Skin
Ninohe	0.074	0.012	0.009	0.004	0.004	0.006	0.006	0.005	0.007	0.003	0.001	0.003	0.000	0.001
Yokote	0.099	0.022	0.009	0.010	0.003	0.004	0.008	0.004	0.006	0.005	0.004	0.003	0.001	0.000
Saku	0.081	0.014	0.009	0.007	0.007	0.005	0.006	0.005	0.006	0.004	0.003	0.003	0.001	0.000
Katsushika	0.102	0.024	0.011	0.007	0.006	0.009	0.006	0.008	0.007	0.004	0.003	0.002	0.002	0.000
Ishikawa	0.075	0.009	0.011	0.003	0.004	0.004	0.004	0.011	0.003	0.003	0.008	0.007	0.002	0.000
Kasama	0.104	0.032	0.008	0.008	0.006	0.004	0.010	0.010	0.004	0.003	0.003	0.002	0.001	0.001
Kashiwazaki	0.106	0.027	0.008	0.010	0.004	0.008	0.007	0.001	0.010	0.010	0.006	0.002	0.000	0.000
Tosayamada	0.094	0.023	0.010	0.005	0.004	0.006	0.003	0.009	0.008	0.003	0.003	0.004	0.001	0.000
Arikawa	0.105	0.018	0.011	0.006	0.014	0.006	0.007	0.005	0.005	0.004	0.005	0.006	0.003	0.001
Miyako	0.079	0.011	0.012	0.005	0.006	0.004	0.009	0.004	0.003	0.003	0.004	0.007	0.001	0.000
Suita	0.102	0.021	0.011	0.007	0.009	0.009	0.006	0.008	0.004	0.003	0.003	0.004	0.001	0.000

SMRs for site-specific cancer mortality rates are shown in Tables 12 and 13. Site-specific SMRs varied widely within the areas; more than 70% of participants had SMR over 110 (high) or under 90 (low).

**Table 12.** Standardized mortality ratio (SMR) for each are for male.

HC	All cancer	Stomach	Lung	Liver	Pancreas	Colon	Esophagus	Rectum	Biliary tract	Leukemia	Lymphoma	Prostate	Skin
Ninohe	84.9	74.7	101.6	45.2	117.4	90.8	36.8	119.8	132.8	49.2	54.0	160.3	124.0
Yokote	105.8	132.8	103.5	47.6	98.9	103.1	198.9	69.4	136.3	84.1	34.1	86.2	89.6
Saku	91.3	97.4	87.4	39.1	111.0	110.9	130.8	113.6	98.3	93.7	115.0	120.4	42.3
Katsushika	109.1	117.7	104.7	97.9	104.0	124.8	139.2	106.5	83.1	97.9	84.6	120.9	152.7
Ishikawa	79.9	44.3	102.1	35.7	60.3	110.7	164.5	50.1	41.6	216.0	126.6	92.0	125.6
Kasama	100.7	124.4	84.7	64.5	86.6	91.8	162.0	124.5	80.3	73.8	61.2	107.6	115.5
Kashiwazaki	75.9	97.5	68.4	38.5	50.0	39.7	114.9	81.2	72.8	111.3	30.9	51.7	433.2
Tosayamada	85.6	88.4	92.5	80.3	41.6	57.4	40.1	79.4	79.5	146.3	81.2	134.1	112.4
Arikawa	134.5	99.9	139.0	187.1	110.6	97.7	126.6	98.7	239.6	165.0	224.5	86.3	66.0
Miyako	95.3	71.3	100.8	68.6	93.2	111.1	202.2	94.4	89.4	145.6	92.6	60.5	279.0
Suita	105.8	95.3	108.4	135.5	99.6	125.9	80.1	101.4	102.3	106.7	99.1	100.9	37.5

**Table 13.** Standardized mortality ratio (SMR) for each are for female.

HC	All cancer	Stomach	Lung	Colon	Liver	Breast	Biliary tract	Uterine	Pancreas	Rectum	Leukemia	Lymphoma	Esophagus	Skin
Ninohe	76.7	53.4	82.3	72.2	62.7	92.5	83.9	59.2	107.4	93.0	41.4	101.8	51.4	145.5
Yokote	105.8	122.3	93.6	130.6	51.4	64.8	128.9	65.1	115.0	114.6	103.5	99.1	76.0	55.0
Saku	94.4	104.2	98.3	124.0	86.9	85.2	99.1	76.1	79.5	98.6	85.6	83.1	70.1	99.2
Katsushika	108.7	109.2	119.2	113.9	97.7	132.5	100.2	107.6	112.1	101.1	96.6	87.1	131.4	84.0
Ishikawa	79.6	40.4	146.4	65.9	52.4	48.6	85.2	135.6	54.9	84.9	213.5	187.6	90.7	56.5
Kasama	103.2	128.4	89.1	94.1	79.5	58.5	138.8	155.7	67.0	86.7	85.1	83.1	63.3	65.5
Kashiwazaki	105.1	121.0	93.2	114.9	65.1	93.6	105.3	15.6	109.9	176.4	243.6	42.9	56.9	0.0
Tosayamada	102.6	105.8	85.3	109.2	72.9	91.4	80.8	119.7	160.1	66.5	170.1	104.1	58.0	232.5
Arikawa	110.7	80.4	140.0	117.1	161.5	83.8	114.7	92.4	104.8	91.5	164.0	210.7	130.8	140.8
Miyako	85.9	45.8	140.9	76.5	98.4	47.7	140.3	74.9	76.1	92.5	116.6	176.3	52.3	141.7
Suita	106.7	95.2	126.4	99.9	121.2	120.0	85.8	113.2	105.9	94.3	101.8	116.1	92.1	78.0

## Discussion

The different aspects of each study area may reveal relationships between the different natural environments, individual cultural aspects, or habitual lifestyles. Cross-sectional or ecological studies would be possible across these 11 public health center districts.

To investigate the relationship between exposure to environmental factors and mortality, substantial variation is needed in mortality rates between areas, especially when investigating each cancer site separately. Variability was examined by age-adjusted mortality rates and SMR. Although age-adjusted mortality rates are superior to SMRs in terms of the comparability between areas, they may not be stable when the number of site-specific cancer deaths in an age strata is low.

Variability of site-specific cancer mortality rates in the study areas was compared with corresponding rates of prefectures in Japan (4). Most of the maximum and the minimum values in our study areas were of equal size to those of prefectures, and some values even surpassed the prefecture values. The differences and ratios between maximum and minimum age-standardized mortality rates showed that there were relatively large differences in mortality rates due to stomach, lung, and liver cancers in males and stomach cancer in females. Ratios were relatively large for skin, lymphoma, biliary tract, esophagus, and liver cancers in males and skin and uterine cancers and leukemia in females. The pattern of the ratios was similar to the difference of SMRs. Large SMR differences tended to be seen in large mortality rates and large ratios tended to be seen in small rates. The large value of ratio of age-standardized rates and difference of SMR should be cautiously interpreted, however, because they were heavily influenced by minimum values and absolute numbers. Due to these reasons, in addition to their public health impact, absolute rates and differences should be taken into account in etiological studies..

In summary, the study areas of the JPHC Study showed different patterns of SMR for major causes of death. And, for most of the sites examined in this paper, there appear to be sufficient differences in site-specific cancer mortality rates to draw inferences regarding possible environmental, cultural, and lifestyle causal factors..

## References

[1] Statistics Bureau, Management and Coordination Agency. Population of Japan: final report of the 1990 population census. 1990.

[2] Statistics and Information Department, Minister’s Secretariat, Ministry of Health and Welfare. Health center- and local municipality-specific vital statistics: special report on vital statistics. 1988-1992.

[3] International statistical classification of diseases, injuries, and causes of death, 9th rev. Geneva: World Health Organization, 1977.

[4] Statistics and Information Department, Minister’s Secretariat, Ministry of Health and Welfare. Age-adjusted death rates by prefecture: special report on vital statistics. 1990.

